# Rice flour-based filled hydrogel: an effective vitamin D encapsulation system as influenced by rice flour variety

**DOI:** 10.1007/s10068-024-01806-7

**Published:** 2025-01-07

**Authors:** Chaeyoung Kim, Shinjae Park, Shin-Joung Rho, Yong-Ro Kim

**Affiliations:** 1 Department of Biosystems Engineering, Seoul National University, Seoul, 08826 Republic of Korea; 2 Convergence Major in Global Smart Farm, Seoul National University, Seoul, 08826 Republic of Korea; 3 Center for Food and Bioconvergence, Seoul National University, Seoul, 08826 Republic of Korea; 4 Research Institute of Agriculture and Life Sciences, Seoul National University, Seoul, 08826 Republic of Korea

**Keywords:** Rice flour, Filled hydrogel, Vitamin D, Physicochemical property, Stability

## Abstract

This study investigated the effects of the physicochemical properties of filled hydrogels based on rice flour with varying amylose contents on the stability of encapsulated vitamin D_3_ (VD, cholecalciferol). The filled hydrogel, prepared based on Saemimyeon with an amylose content of 25.5%, formed a gel with strong elasticity, showing the highest stability during in vitro digestion, as well as excellent pH and storage stability of the encapsulated VD. Dodamssal, which had the highest amylose content (> 40%) and gelatinization temperature, retrograded rapidly due to its high leached amylose content. This phenomenon caused gel shrinkage and subsequent emulsion leakage, resulting in a relatively lower VD retention rate after in vitro digestion compared to the Saemimyeon-based filled hydrogel. Nevertheless, compared to the emulsion system, the rice flour-based filled hydrogels formed various types of gel matrices that provided a physical barrier to the VD-containing lipid droplets, thereby significantly protecting them from the external environment.

**Supplementary Information:**

The online version contains supplementary material available at 10.1007/s10068-024-01806-7.

## Introduction

Emulsions are effective drug delivery systems that encapsulate functional substances, thereby controlling release rates and enhancing bioavailability (Ezhilarasi et al., 2013). However, oil-in-water (O/W) emulsions are prone to both thermodynamic and chemical instability during processing, storage, and transportation, mostly through oxidative reactions (Calero et al., 2013). To address this, novel hydrogel systems that combine polysaccharides and emulsions have gained considerable attention (Calero et al., 2013). These systems are considered ideal for related applications due to their rheological properties, capability to effectively encapsulate nanosized droplets within the gel matrix, and their potential for co-delivering enhancers within the gel matrix (Light and Karboune, 2022). The three-dimensional gel matrix of the hydrogel, combined with the emulsions, can enhance their mechanical behavior, providing physical protection and controlling the release of hydrophobic bioactive compounds within the emulsion (Coviello et al., 2007). Polysaccharide-based hydrogels have received considerable attention due to their promising properties for biomaterial development, including biocompatibility, non-toxicity, and susceptibility to microbial degradation (Coviello et al., 2007; Reis et al., 2008).

Rice flour, which contains a significant amount of starch, can be applied as a natural hydrogel material by forming a three-dimensional gel network via gelatinization and retrogradation, due to its high starch content. Rice, a major staple food in Asia, is extensively used in various rice-based processed foods such as rice cakes, rice noodles, and bread, contingent upon the cultivar (Hong et al., 2021). Moreover, rice-based gel products are gaining increased acceptance worldwide (Houjyo et al., 2017). The amylose content in starch, the main component of rice flour, affects its gelatinization and retrogradation properties (Sasaki et al., 2000). A high amylose content promotes retrogradation, leading to the formation of a more organized and rigid crystalline structure. Consequently, starch hydrogels with high amylose content may form hard gels with greater swelling resistance and mechanical strength, reducing their water absorption capacity (Biduski et al., 2018). In contrast, hydrogels with relatively low amylose contents exhibit an open structure that makes them prone to disintegration in water. These properties contribute to the formation of various three-dimensional network structures in emulsions, thereby affecting emulsion stability (Houjyo et al., 2017).

Filled hydrogels based on rice starch can be used to protect various hydrophobic substances encapsulated in emulsions. Mun et al. (2015) and Kim et al. (2023) confirmed that the bioaccessibility of beta-carotene was improved by combining an emulsion with rice starch or starch blend hydrogels. Kang et al. (2021) suggested that a filled hydrogel based on enzyme-modified rice starch could improve the chemical stability of curcumin in emulsions by providing a physical protective layer. However, despite the economic advantage of rice flour being directly applicable to food processing compared to rice starch, research on using rice flour as a filled hydrogel is still lacking.

In this study, vitamin D_3_ (VD, cholecalciferol), was used as the bioactive compound for encapsulation. VD is an essential hydrophobic micronutrient for maintaining health and well-being, particularly in older populations that are prone to VD deficiency (Liu et al., 2020). While VD can be consumed in limited quantities in foods such as beef liver, egg yolk, and dairy products, achieving sufficient intake levels can be challenging, necessitating the development of functional foods in diverse formulations. Additionally, VD is highly vulnerable to environmental factors such as light, heat, and oxygen exposure, which can lead to rapid decomposition and loss of biological activity, thereby limiting its utility in food applications (Temova Rakuša et al., 2021).

Based on these considerations, the objective of this study was to investigate the effects of the gel network properties of rice flour with various amylose contents used as hydrogel on the stability and bioaccessibility of encapsulated VD in the filled hydrogel. For this purpose, four rice flour varieties (Dodamssal, Saemimyeon, Saeilmi, and Mirchal) of different amylose contents were used to prepare rice flour-based filled hydrogels encapsulating VD, and the relationship between physicochemical properties of rice flour gels and VD encapsulating performance was investigated. The results of this study are promising for the development of novel VD-enriched food materials based on rice flour gel matrices, particularly for those who are vulnerable to VD deficiency.

## Materials and methods

### Materials

Korean rice cultivars, Dodamssal, Saemimyeon, Saeilmi, and Mirchal, were provided by Department of Central Area Crop Science of National Institute of Crop Science (Rural Development Administration, Suwon, Korea) in the form of dry milled rice flour with a D50 particle size range of 75–80 μm. The provided rice flours were used after sieving through a 100-mesh sieve (Chunggye Inc., Seoul, Korea). Whey protein isolate and soybean oil were obtained from Protient Inc. (Saint Paul, MN, USA) and Ottogi Corp. (Pyeongtaek, Korea), respectively. Vitamin D_3_ (VD, cholecalciferol, ≥ 98% purity) was purchased from Sigma-Aldrich (St. Louis, MO, USA). All chemicals and reagents used were of analytical grade.

### Physicochemical properties of rice flours

The apparent amylose content (%) of rice flour was measured using the AACC (2000) method. Total digestible starch and resistant starch contents were determined using a Megazyme K-DSTRS assay kit (Megazyme Ltd., Wicklow, Ireland). Rice flours were incubated with a mixture of pancreatic α-amylase and amyloglucosidase (PAA/AMG) and subsequently hydrolyzed with AMG (100 U/mL). Residues were measured using the GOPOD (glucose oxidase–peroxidase and 4-aminoantipyrine) reagent enzyme. Swelling power (g/g) and water solubility index (%) of rice flours were measured using a modified method from a previous study (Hong et al., 2021).

Gelatinization and retrogradation thermal profiles of rice flours were performed using a differential scanning calorimeter (DSC, Perkin-Elmer DSC 4000, Norwalk, CT, USA). Samples (rice flour: distilled water = 1:3, dry basis) were heated from 30 to 120°C at a rate of 10°C/min. Subsequently, they were stored at 4°C for 2 and 4 weeks, then reheated from 10°C to 100°C at a rate of 10°C/min.

### Preparation of the filled hydrogel

Filled hydrogels were prepared using the method described by Kim et al. (2023), with slight modifications. The oil phase of the oil-in-water (O/W) emulsion was prepared by sonicating soybean oil containing VD (0.2%, w/w) for 5 min using an ultrasonic cleaner (Powersonic 410, Hwashin Tech Co. Ltd., Seoul, Korea) and then heating the mixture at 60°C for 30 min. The aqueous phase was prepared by dissolving whey protein isolate (0.5%, w/w) in 5 mM phosphate buffer (pH 7) for 30 min with stirring. A coarse emulsion (5% oil, w/w) was prepared by homogenizing the oil and aqueous phase solutions with a high-speed blender (ULTRA-TURRAX model T25 digital, IKA, Germany) for 2 min, followed by processing through a microfluidizer (Picomax MN 250A, Micronox, Seongnam, Korea). The rice flour-based filled hydrogel was prepared by dispersing rice flour (10%, w/w) into the O/W emulsion at 90°C for 10 min, and then cooling it to room temperature (24°C).

### Characteristics of the filled hydrogel

The rheological behaviors were determined with a rheometer (AR 1500 ex, TA Instruments Ltd., New Castle, USA) using a 20 mm parallel plate geometry at a gap of 1 mm. Frequency sweep tests were performed at a frequency range of 0.1 to 10 Hz at a strain of 0.5%. The storage modulus (G′), loss modulus (G″), and tan δ (G″/G′) were determined and plotted as functions of frequency. Data analysis was performed using the TA rheometer software (Version V.4.20, TA Instruments Inc., New Castle, DE, USA). Frequency (f) dependence of the storage modulus (G′) was fitted to power-law model equations (Eq. 1):

1 $$G{\prime}=K\times {f}^{n}$$

wher*e K* is the power-law modulus constant and *n* represents the frequency modulus exponents.

Freeze–thaw stability was performed according to our previous research method (Hong et al., 2021). Filled hydrogel samples were frozen in a freezer at -18°C for 22 h and thawed at an incubator at 25°C for 2 h. This freeze–thaw cycle was repeated five times. The measured weight (W_a_) of the thawed filled hydrogel was placed in a cylindrical tube with a 60-mesh sieve and a Whatman No. 2 filter paper attached to the bottom. Afterwards, they were centrifuged at 100 ×g for 15 min at 25°C using a centrifuge (Supra 22 K, Hanil Science Inc., Incheon, Korea). The cylindrical tube containing the hydrogel was removed, and the remaining liquid in the conical tube was weighed (W_b_). Syneresis (%) was calculated by dividing the liquid mass (W_b_) separated from the filled hydrogel during centrifugation by the initial mass (W_a_) of the filled hydrogel before centrifugation, and then multiplying by 100.

The cross-sectional structure of the filled hydrogel was observed by field-emission scanning electron microscopy (FE-SEM, SUPRA 55VP, Carl Zeiss, Germany). All samples were lyophilized using a freeze dryer (FD8508, Ilshin BioBase, Dongducheon, Korea) until completely dehydrated, then sectioned into approx. 1 mm thick slices. Imaging was performed at 100× and 250× magnifications at 2.00 kV.

The X-ray diffractograms of the lyophilized filled hydrogel samples were measured at 2*θ* angles ranging from 5° to 40°, with a step size of 0.02° and a scanning speed of 0.5°/min using an X-ray diffractometer (XRD, D8 Advance with Davinci, Bruker, Germany).

### Stability of VD in the filled hydrogel

Evaluation of stability of encapsulated VD against storage and heat was determined by storing filled hydrogel and emulsion samples at 4°C, 25°C, and 40°C for 28 days in the dark. To measure pH stability, samples (25%, w/v) were prepared by adding them to buffer solutions adjusted to pH 2 and pH 11 with HCl and NaOH, respectively, for 24 h. The retention rate of remaining VD in samples treated under various conditions was adopted from a previous study (Mekala et al., 2022) with slight modification. All samples were blended with chloroform/methanol (2:1, v/v) and centrifuged at 12000 ×g for 10 min. After the bottom layer was collected and redissolved in chloroform, retention rate (%) of VD remaining undegraded was measured using a UV/Vis-spectrophotometer (UV-1650 PC, Shimadzu, Tokyo, Japan) at 266 nm.

### In vitro digestion via simulated gastrointestinal tract (GIT) model

In vitro digestibility test was conducted using a simulated gastrointestinal tract (GIT) model consisting of mouth, stomach, and intestinal phases as described in our previous study (Kim et al., 2023; Mun et al., 2015). Lipid digestibility was measured by recording the pH of the mixture and measuring the volume of 0.25 M NaOH required to neutralize the free fatty acids released during the lipid digestion over 2 h. After in vitro digestion, raw digesta were mixed with chloroform and centrifuged (543 ×g, 10 min) to extract the VD, which was then analyzed using a UV–Vis spectrophotometer at 266 nm.

### Confocal laser scanning microscopy (CLSM)

Samples were stained with Nile red (0.1% w/v in ethanol), and structural changes in lipid droplets at each phase (initial, mouth, stomach, and intestine) were observed using confocal laser scanning microscopy (CLSM, Leica TCS SP8 X, Leica Microsystems, Wetzlar, Germany).

### Statistical analysis

Each experimental measurement was performed in triplicate using three independent replicates of samples. Data were expressed as the mean ± standard deviation. Statistical analysis was performed using SPSS (IBM SPSS Statistics for windows, Version 25.0, Armonk, NY, USA). One-way ANOVA was used for comparing group means, followed by Duncan’s multiple range test to determine the significance of individual comparisons (*p* < 0.05).

## Results and discussion

### Physicochemical properties of rice flours

#### In vitro digestibility and hydration properties of rice flours

**Fig. 1:**
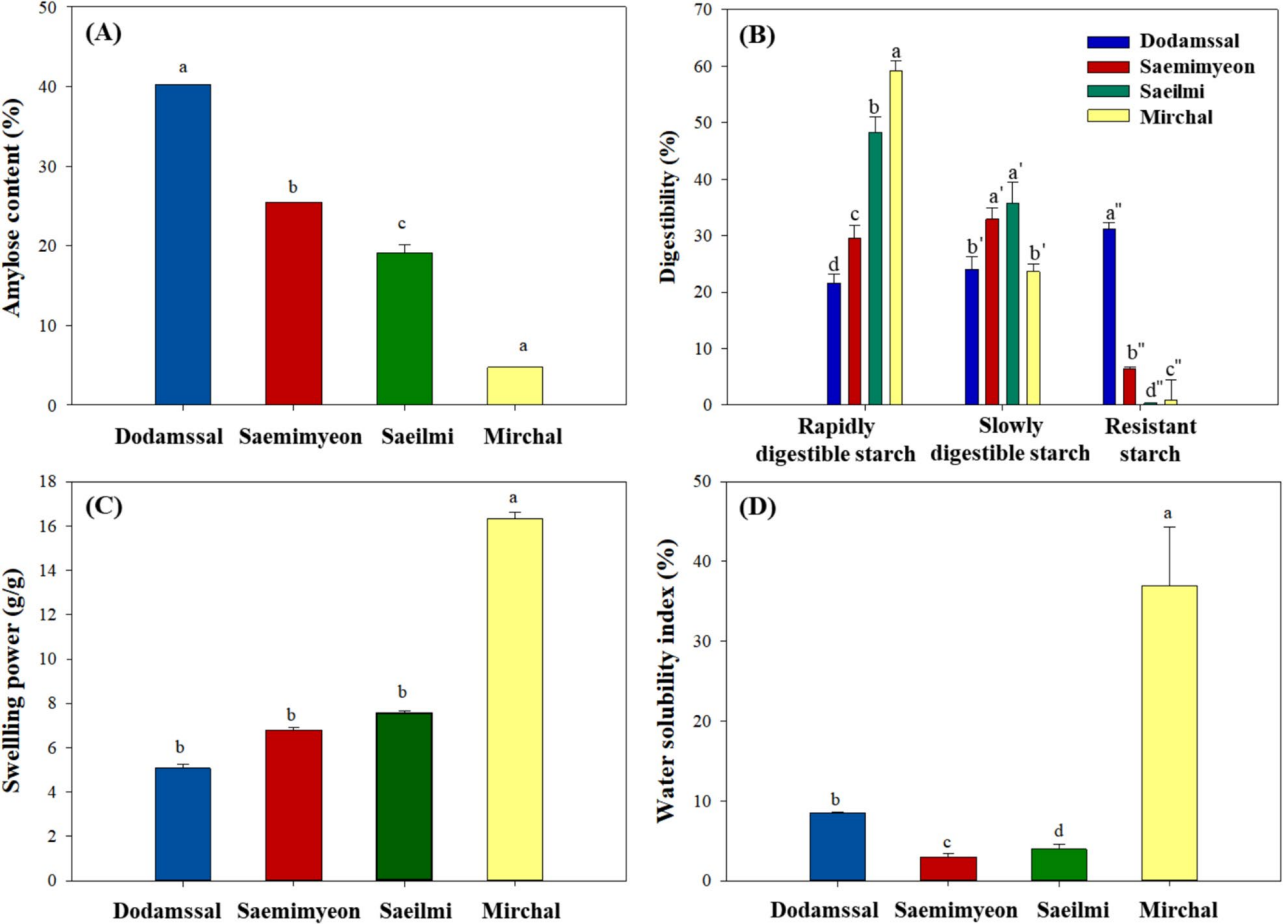
**A** Apparent amylose content and (**B**) rapidly digestible starch, slowly digestible starch, and resistant starch of rice flours. **C** Swelling power and (**D**) water solubility index of various rice flours at 80°C. Values represent means ± standard deviation of triplicate experiments. Results marked with different letters above the bars indicate significant differences (*p* < 0.05)

The amylose contents of the four rice flour varieties were in the following order: Dodamssal (40.2%) > Saemimyeon (25.5%) > Saeilmi (19.1%) > Mirchal (4.7%) (Fig. 1A). The amount of rapidly digestible starch in the rice flour decreased as the apparent amylose content of rice flour increased, whereas resistant starch had the opposite trend (Fig. 1B). These results are consistent with previous studies that indicated a positive correlation between amylose content and resistant starch content in granular starches (Zhu et al., 2011). Amylose molecules in amorphous regions are initially hydrolyzed by amylases but later associate and resist enzymatic digestion (Zhu et al., 2011).

The swelling power (g/g) and water solubility index (%) of starch indicate the ability of starch granules to absorb water and the extent of amylose leaching out during swelling, respectively (Farooq et al., 2021). Rice flours with relatively high amylose and resistant starch contents had lower swelling power (Fig. 1C), which was consistent with the results of a previous report (Zhang et al., 2017). Starches with high amylose contents may structurally hinder water uptake due to internal hydrogen bond formation between amylose chains (Zhang et al., 2017). Conversely, waxy starch with a low amylose content has a high swelling power due to its open structure, which allows rapid water penetration.

The water solubility index of a rice flour indicates its ability of solids to dissolve or disperse in an aqueous solution, and is related to the presence of soluble molecules such as amylose (Farooq et al., 2021). Mirchal rice flour, which has a low amylose content, had significantly higher water solubility index values due to relatively low leaching of amylose (Fig. 1D). Dodamssal, a rice flour with a high gelatinization temperature, prevented amylose from leaching due to incomplete gelatinization at 80°C, resulting in a higher water solubility index value than those of Saemimyeon and Saeilmi rice flours.

#### Changes in the thermal properties of gelatinized rice flours after retrogradation

The thermal properties of rice flours, as measured by DSC for gelatinization and retrogradation, are presented in Table 1. The highest observed onset temperature (T_o_), peak temperature (T_p_), and conclusion temperature (T_c_) values were in the order of Dodamssal, Saemimyeon, Saeilmi, and Mirchal, in agreement with the previously mentioned resistant starch contents. T_o_ refers to the specific temperature at which the thermal transition begins, indicating the point at which starch granules start absorbing heat and water. Taken together, these results suggest that the presence of resistant starch may delay the initiation of gelatinization. da Rosa Zavareze et al. (2012) reported that T_o_, T_p_, and T_c_ values increased consistently with higher resistant starch content under heat-moisture treatment. The presence of resistant starch significantly enhances the thermal and shear stability of the granules, thereby requiring higher temperatures and longer durations for the onset of gelatinization (Sharma et al., 2015). This observation is supported by the swelling power results (Fig. 1C), wherein Dodamssal exhibited significantly lower swelling power compared to Mirchal, indicating a relatively reduced moisture absorption rate. This implies that the T_o_ for Dodamssal is likely to be higher. In contrast, there was no significant difference in the *Δ*H values among the rice cultivars. The amount of heat absorbed during the gelatinization process, *Δ*H, serves as an indicator of the energy required to disrupt the crystalline regions within the starch granules (Kong and Hay, 2002). Therefore, the degree of crystallinity among the cultivar is likely similar until retrogradation occurs.

**Table 1:** Changes in thermal properties of rice flours during 2 and 4 weeks of storage

Conditions	Rice flours	T_o_ (℃)^1^	T_p_ (℃)	T_c_ (℃)	*Δ*H (J/g)	*Δ*T
Gelatinization	Dodamssal	68.61 ± 0.48^a^	79.12 ± 0.71^a^	87.87 ± 1.09^a^	6.28 ± 0.06^b^	19.26 ± 0.63^a^
	Saemimyeon	68.74 ± 0.26^a^	73.67 ± 0.10^b^	79.60 ± 0.12^b^	7.18 ± 0.76^a^	10.86 ± 0.38^d^
	Saeilmi	61.43 ± 0.07^b^	67.51 ± 0.24^c^	75.47 ± 0.38^d^	6.85 ± 0.10^ab^	14.04 ± 0.31^c^
	Mirchal	60.36 ± 0.45^c^	67.92 ± 0.55^c^	76.83 ± 0.65^c^	7.63 ± 0.34^a^	16.48 ± 0.53^b^
Dissociation of retrograded rice flour (2 weeks)	Dodamssal	43.76 ± 1.11^a^	60.15 ± 1.32^a^	71.68 ± 1.09^a^	1.73 ± 0.27^b^	27.92 ± 2.17^a^
	Saemimyeon	39.58 ± 1.23^b^	52.37 ± 2.92^b^	62.14 ± 1.28^b^	3.94 ± 0.34^a^	22.56 ± 0.92^b^
	Saeilmi	40.57 ± 0.04^b^	50.50 ± 0.21^b^	58.41 ± 1.10^c^	1.13 ± 0.35^b^	17.84 ± 1.13^c^
	Mirchal	ND^2^	ND	ND	ND	ND
Dissociation of retrograded rice flour (4 weeks)	Dodamssal	40.50 ± 0.89^b^	59.75 ± 0.55^a^	72.78 ± 1.06^a^	2.66 ± 0.51^b^	32.28 ± 1.81^a^
	Saemimyeon	38.86 ± 0.60^c^	50.65 ± 0.69^b^	62.06 ± 0.50^b^	3.72 ± 0.04^a^	23.20 ± 1.08^b^
	Saeilmi	41.98 ± 0.45^a^	51.16 ± 0.52^b^	57.62 ± 0.92^c^	2.57 ± 0.23^b^	15.65 ± 1.36^c^
	Mirchal	ND	ND	ND	ND	ND

^1^T_o_, onset temperature; T_p_, peak temperature; T_c_, temperature at the end of melting; *Δ*H, enthalpy of melting; *Δ*T, melting range

^2^ND: not detected

Values represent means ± standard deviation of triplicate experiments. Results in the same column of sample groups with different letters (a-d) are significantly different (*p* < 0.05)

When the retrograded rice flours were reheated after being stored at 4°C for 2 or 4 weeks, the transition temperatures and *Δ*H values decreased compared to the initial value. This indicated the formation of incomplete crystallization and a less stable structure resulting from retrogradation (Fu et al., 2013). For Mirchal, a waxy rice flour, no retrogradation peak was observed in the DSC analysis. The limited leaching of amylose in rice flour with low amylose content may not allow re-crystallization of amylopectin molecules (Zhou et al., 2010). Generally, the retrogradation rate of rice starch is known to be related to amylose content (Fu et al., 2013). However, Dodamssal showed a lower *Δ*H value than Saemimyeon after retrogradation for both 2 and 4 weeks, which may be attributed to limited hydration and swelling under the gelatinization conditions used in this study. After retrogradation, the *Δ*H value of Saemimyeon was relatively higher than that of other rice varieties, suggesting the formation of a more extensive recrystallized amylopectin structure (Li et al., 2022).

##### Characteristics of the filled hydrogels

#### Rheological properties

**Fig. 2:**
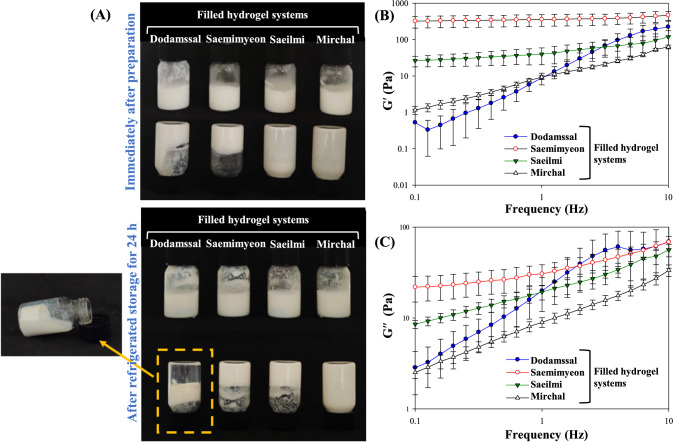
**A** The visual appearance of the prepared rice flour-based filled hydrogel at the initial stage (the upper part) and after 24 h of refrigerated storage (the lower part) and (**B**) storage modulus (G′) and (**C**) loss modulus (G′′) of filled hydrogels at 0.1–10 Hz frequency sweep test

After refrigerating the rice flour-based filled hydrogels for 24 h, the flowability of the gel was visually observed by inverting a vial (Fig. 2A). Mirchal-based filled hydrogel was observed to flow in a sol state, without gel formation, even after 24 h. The Saeilmi-based filled hydrogel exhibited flow immediately after preparation, but then transitioned to a gel state on refrigeration, preventing further flow. The Saemimyeon-based filled hydrogel exhibited rapid gel formation due to retrogradation both before and after storage. The Dodamssal-based filled hydrogel flowed initially, but formed a weak gel after refrigeration. However, noticeable coalescence of the leaked emulsion due to shrinkage of the formed gel during storage was observed.

The rheological properties of the Saemimyeon- and Saeilmi-based filled hydrogels showed solid-like behavior, typical of semi-solid foods, with G′ consistently higher than G″ (Fig. 2B and Table S1). Notably, the filled hydrogel prepared with Saemimyeon, which has a high amylose content, formed a hard gel compared to those prepared with the Saeilmi and Mirchal, which have relatively low amylose content. The higher amylose content in rice flour can hydrogen bond with each other to form a relatively rigid gel (Hong et al., 2021). Despite being prepared from high amylose content Dodamssal, the Dodamssal-based filled hydrogel initially showed a rapid increase in both G' and G'', these values subsequently decreased due to incomplete swelling of granules and gel shrinkage being reduced by rapid retrogradation (Figs. 2B and C). The liquid that did not gel and separated in Dodamssal based filled hydrogel due to retrogradation appears to have affected the G' and G'' values, resulting in liquid-like characteristics (tan *δ* value > 1) (Table S1). In contrast, the Saemimyeon and Saeilmi-based filled hydrogels had *n* values below 0.5, indicating frequency independence and, possibly, the formation of gels with solid-like characteristics (Zhang et al., 2017). The characteristics of Dodamssal and Mirchal-based filled hydrogels were closer to those of a sol; gel network formation was incomplete in the Dodamssal-based filled hydrogel due to the rapid shrinkage and syneresis associated with retrogradation.

#### Freeze–thaw stability

**Fig. 3:**
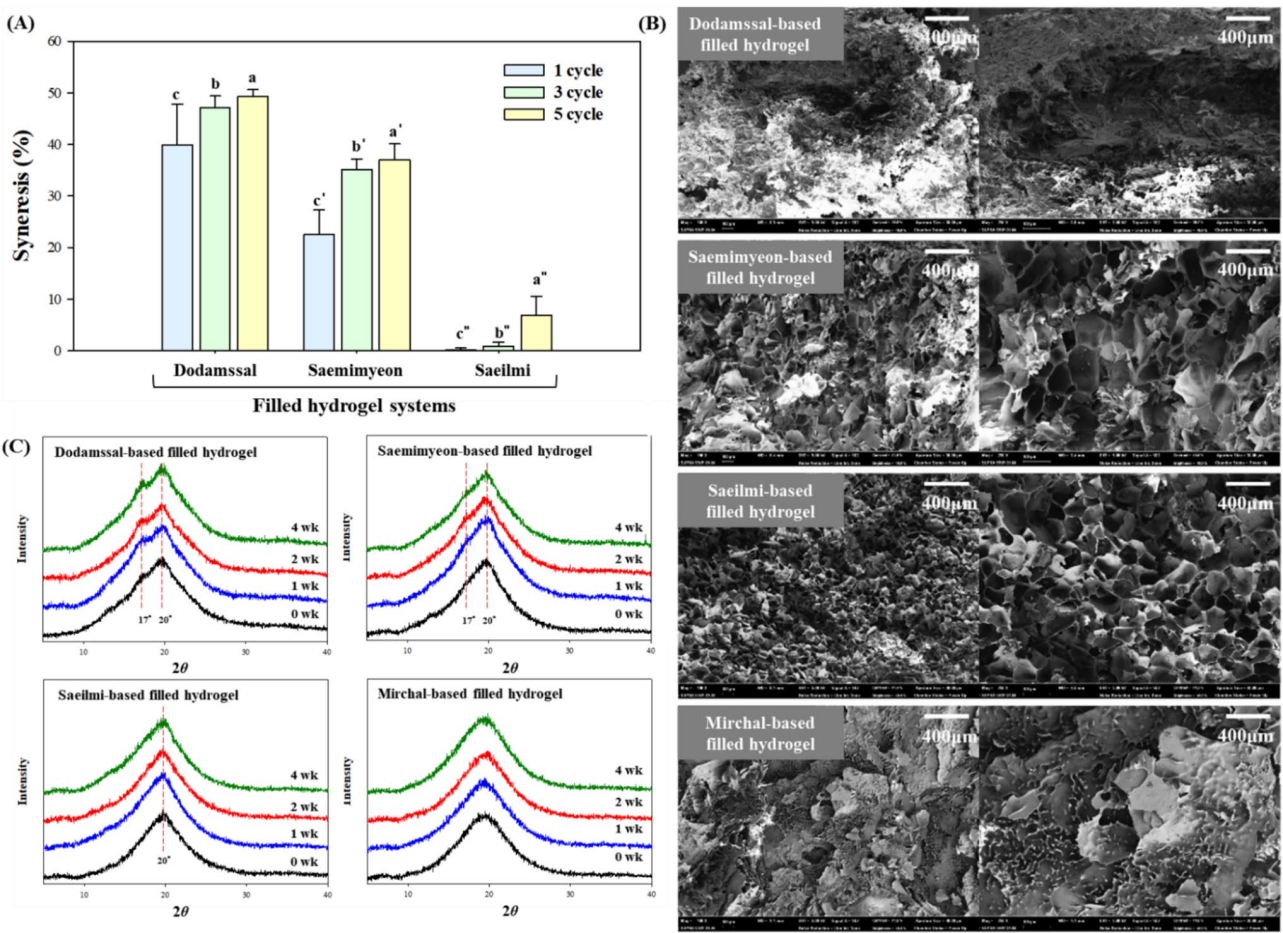
**A** Syneresis of filled hydrogels during 1, 3, 5 freeze–thaw cycles, (**B**) microstructure of filled hydrogels cross–section (magnification: ×100 on the left and ×250 on the right), **C** X-ray diffraction patterns of filled hydrogels during 4 weeks of storage. Values represent means ± standard deviation of triplicate experiments. Results marked with different letters above the bars indicate significant differences (*p* < 0.05)

Syneresis, a significant parameter for characterizing the freeze–thaw stability of hydrogel systems, indicates the ability of hydrogel to withstand physical changes during freeze–thaw treatments (Hong et al., 2021). The syneresis of all samples significantly increased with repeated freeze–thaw cycles compared to the first cycle (Fig. 3A). An increased number of freeze–thaw cycles can lead to greater retrogradation and phase separation of gels and pastes composed of starch, ultimately resulting in the formation of a sponge–like structure that potentially increases syneresis (Yuan and Thompson, 1998). Filled hydrogels prepared from Dodamssal and Saemimyeon, with high amylose content, resulted in high syneresis values across all freeze–thaw cycles. This result is consistent with those of previous studies indicating a negative correlation between amylose content and freeze–thaw stability (Puncha-arnon et al., 2008). Consistent with this, the Mirchal-based filled hydrogel showed no syneresis attributed to retrogradation throughout all freeze–thaw cycles.

#### Cross-sectional structure of the filled hydrogels

The cross-sectional structure of the Dodamssal-based filled hydrogel could not be clearly visualized due to image charging (Fig. 3B). In contrast, both the Saemimyeon and Saeilmi-based filled hydrogels showed distinct and clear porous network structures. Previous studies indicated that a high amylose content could impede water release during freeze-drying by intensifying hydrogen bond interactions among starch chains. Moreover, a high amylose content contributes to increased susceptibility to retrogradation, leading to the development of pores outside the hydrogel structure (Biduski et al., 2018). The Saeilmi-based filled hydrogel displayed a more compact structure compared to Saemimyeon-based filled hydrogel, influenced by amylose-induced retrogradation. Retrogradation, which promotes the formation of new hydrogen bonds, can enlarge pore size and lead to syneresis (Yuan and Thompson, 1998). Therefore, this observation is consistent with the results for freeze–thaw stability results, indicating that the Saemimyeon-based filled hydrogel with its relatively high amylose content exhibits more syneresis than the Saeilmi-based filled hydrogel. The Mirchal-based filled hydrogel lacked observable pore formation; instead, it displayed continuous phases that formed a broad sheet structure. This suggests that the Mirchal-based filled hydrogel existed in a sol state, where gelation did not occur after gelatinization, consequently lacking the typical pore structure of the gel.

#### Changes in the crystallinity of the filled hydrogels during storage

The XRD patterns and crystallinity of the filled hydrogels are shown in Fig. 3C. Before storage, with the exception of the Mirchal-based filled hydrogel, all samples indicated a V-type diffraction pattern with a pronounced peak at a diffraction angle (2*θ*) of 20°. This indicated the formation of an amylose–lipid complex, involving amylose from rice flour and fatty acids and phospholipids from the emulsion (Tian et al., 2010). The Mirchal-based filled hydrogel, with its low amylose content of < 5%, did not exhibit the distinct peak associated with amylose–lipid complexes.

After storage, rapid retrogradation occurred in the Dodamssal and Saemimyeon-based filled hydrogels due to amylose leaching and subsequent amylopectin reassembly. These hydrogels showed a B-type crystalline structure, with a diffraction peak at 2*θ* = 17°, due to retrogradation after storage (Ma et al., 2019); the peak intensity increased with the storage time. The crystal strength of the Dodamssal-based filled hydrogel was higher than that of the Saemimyeon-based filled hydrogel, which indicates that although it did not produce high-quality crystals, the perfectness or orderness of the crystals was much higher. In contrast, the filled hydrogels prepared with the Saeilmi and Mirchal, which have relatively low amylose contents, did not show a peak at 17°. This lack of peak formation is attributed to hindered recrystallization due to the reversible and long-term retrogradation process of amylopectin compared to amylose. Additionally, lipids in the filled hydrogel may form complexes with the outer branches of amylopectin, further inhibiting the recrystallization of both amylose and amylopectin (Chumsri et al., 2022).

### Stability of VD encapsulated in the filled hydrogels and emulsion

#### Storage and heat stability

**Fig. 4:**
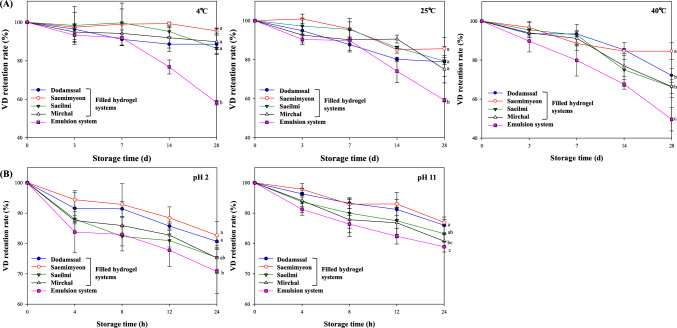
Retention rate of vitamin D (VD) encapsulated in filled hydrogel and emulsion systems (**A**) at 4, 25, and 40°C during 28 days of storage and (**B**) at pH 2 and pH 11 during 24 h of storage

As vitamin D_3_ (VD, cholecalciferol) in food is easily destroyed during heat treatment and storage, maintaining its stability is a very important during food processing such as cooking or sterilization (Mahmoodani et al., 2017). VD may undergo reversible isomerization to pyrocholecalciferol and isopyrocholecalciferol, depending on the temperature and time of exposure (Mahmoodani et al., 2017). To evaluate the stability of VD encapsulated in emulsion and filled hydrogel samples, the amounts of VD remaining (retention rate, %) were measured after storage at 4°C, 25°C, and 40°C for 4 weeks (Fig. 4A). Degradation of encapsulated VD increased with higher storage temperatures, and the VD retention rates were significantly higher in the filled hydrogels than in the emulsion under all conditions. These findings are attributed to the rice flour acting as a hydrogel matrix, offering protection to VD by creating a barrier against the oxidative degradation induced by exposure to air. Moreover, the compact structure of these hydrogels may slow down oxygen diffusion into the gel (Liu et al., 2020). Notably, even after 4 weeks of storage at 40°C, the Saemimyeon-based filled hydrogel maintained a VD retention rate of over 80%. This may have been be due to the formation of a hard gel with a compact structure and a high G' value (Table S1). The high amylose content of Saemimyeon contributes to accelerated retrogradation, resulting in a more rigid gel that effectively preserves oil droplets and shields the VD from exposure to air. Although the Dodamssal-based filled hydrogel also improved heat and storage stability, it showed a relatively lower VD retention rate than the Saemimyeon-based filled hydrogel. This lower retention rate was likely due to emulsion leakage resulting from the formation of an incomplete gel network.

#### pH stability

The stability of the emulsion formulations used in the food industry is significantly influenced by pH. Because the pH within the digestive tract varies from the highly acidic in the stomach to the neutral to slightly alkaline in the small intestine, assessing pH stability is crucial to understanding emulsion behavior (Liu et al., 2017). VD may decompose in low pH environments, leading to the formation of isomerization products such as isotachysterol (Mahmoodani et al., 2017). Moreover, their degradation can be accelerated by the oxidation of unsaturated lipids (Hemery et al., 2015). As shown in Fig. 4B, the VD in all samples was more rapidly destroyed under acidic conditions (pH 2.0). In the emulsion system, degradation reached approx. 18% after 4 h of storage. A previous study reported that VD stability decreased relatively sharply, especially below pH 5 (Temova Rakuša et al., 2021). While VD did not undergo rapid destruction as at pH 2, gradual degradation occurred even under pH 11 conditions. Throughout the storage period, the VD retention rate was higher in filled hydrogel systems than in the emulsion system. This enhanced stability in the filled hydrogels was likely due to the physical barrier provided by the gelatinized rice flour matrix, which protected the VD from the acidic and alkaline environments. In particular, the Saemimyeon-based filled hydrogel formed a stable gel that resisted complete destruction even after 12 h of physical agitation. In gel formulations, continuous stirring can lead to rapid collapse and dissolution in the solvent, as well as oil droplet release (Guo et al., 2015). However, in hard gels, the reduced particle breakdown inhibits oil release. Thus, the structural characteristics of the gelatinized hydrogels play a critical role in preventing contact between the emulsion within the filled hydrogel and extreme pH conditions, thereby contributing to maintaining a high VD retention rate.

### In vitro digestion test

#### Lipid digestibility

**Fig. 5:**
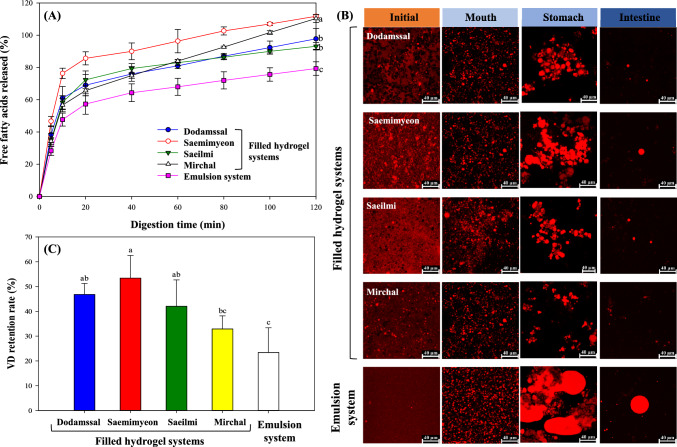
**A** Amounts of free fatty acids released after sample digestion in a pH–stat in vitro digestion model, **B** microstructural changes during in vitro digestion observed by confocal laser scanning microscopy (× 630 magnification), and (**C**) retention rate of VD encapsulated in filled hydrogel and emulsion systems after in vitro digestion. Values represent means ± standard deviation of triplicate experiments. Results marked with different letters above the bars indicate significant differences (*p* < 0.05)

The influence of the filled hydrogels on the digestibility of lipid droplets was determined by measuring the neutralization of free fatty acids released during pancreatic lipase action in the gastrointestinal tract (GIT). The concentrations of free fatty acids generated during the hydrolysis of emulsified lipids were calculated by measuring the volume of sodium hydroxide solution required to maintain a neutral pH throughout the lipid digestion process in the small intestine phase (Liu et al., 2017). The profiles of released free fatty acids over the digestion duration for the filled hydrogel systems are shown in Fig. 5A. Free fatty acids were released rapidly within the initial 20 min, followed by a gradual increase until the completion of digestion. Throughout the 2 h intestinal digestion period, the rate of lipid digestion was observed to be higher in the filled hydrogel system compared to the emulsion system. This is consistent with previous research indicating that free fatty acid production per unit time decreases with increasing droplet size (Majeed et al., 2016), which is associated with the surface area of oil exposed to digestive enzymes. In the filled hydrogel samples, the oil droplets remained relatively uniformly dispersed during the stomach phase without significant coalescence (Fig. 5B). Subsequently, in the intestinal phase, lipid droplets were released due to the decomposition of the gel matrix, which increased the surface area available for lipase action, resulting in an acceleration in the rate of lipid digestion. In contrast, in the emulsion sample, the coalescence and aggregation of oil droplets was promoted in the stomach phase, leading to an increase in droplet size. In the intestinal phase, the surface area available for reaction with lipase was reduced due to highly aggregated lipid droplets, which appeared to result in a relative decrease in the rate of lipid hydrolysis.

#### Changes in microstructure after in vitro digestion

Figure 5B illustrates the microstructural changes of filled hydrogels and emulsion throughout GIT digestion. CLSM images revealed significant lipid droplet aggregation within the mouth, stomach, and intestinal phases. In the initial phase, the emulsion showed small lipid droplets compactly packed, whereas the filled hydrogels showed larger lipid droplets that were somewhat aggregated with hydrogel. Meanwhile, in the case of Dodamssal-based filled hydrogel, non-stained spherical shapes were observed in the initial phase. This may be due to the presence of unswollen starch granules of Dodamssal within the filled hydrogel due to the high gelatinization temperature.

In the mouth and stomach phase, the emulsion showed large lipid droplets with fluidity and high viscosity, whereas the filled hydrogels observed the formation of lipid droplet aggregates. This phenomenon was attributed to depletion flocculation due to coagulation of mucin, an especially a high molecular weight biopolymer, with the gelatinized rice flour gel matrix (Chang and McClements, 2016). The filled hydrogels exhibited the formation of lipid droplet clusters, with varying sizes depending on the types of rice flour gel used. Dodamssal-, Saemimyeon-, and Saeilmi-based filled hydrogels formed larger clustered small lipid droplets, whereas the Mirchal-based filled hydrogel contained dispersed lipid droplets with limited cohesion. This difference was attributed to the weaker gel-forming properties of the Mirchal, which has a lower amylose content compared to the other rice flour varieties. Continuous agitation led to the destruction of the weak gel (closer to sol) structure of the Mirchal-based filled hydrogel, allowing exposure of lipid droplets to digestive fluid under acidic conditions (Salvia-Trujillo et al., 2013). The emulsion clearly contained lipid droplets, and several associated compounds were also observed adhering to the lipids. This can be interpreted as depletion flocculation induced by mucin present in the aqueous phase surrounding the droplets (Chang and McClements, 2016). Furthermore, the emulsion lacking a gelatinized rice flour gel matrix had relatively less interference with the coagulation of lipid droplets compared to the filled hydrogel. This results in facile coalescence, leading to an observed increase in lipid droplet size. After the intestinal phase, lipid droplets were not readily observed in any samples. This was attributed to the continuous dilution throughout each phase and lipid digestion caused by lipase. Taken together, these findings demonstrate that the continuous gel network within the filled hydrogels more effectively inhibited the coalescence of emulsion droplets in the GIT system.

#### Retention rates of encapsulated VD after in vitro digestion

The filled hydrogel samples showed significantly higher retention rates of encapsulated VD after digestion compared to the emulsion, as shown in Fig. 5C. The gelatinized rice flour layer in the filled hydrogels can form a protective coating around the lipid droplets, inhibiting lipase access to the encapsulated lipid (Kim et al., 2023). This layer may also increase aqueous viscosity or gelling compared to the emulsion, thereby reducing the diffusion and mixing of lipase (Mun et al., 2015). As explained in the pH stability experiment, the Saemimyeon-based filled hydrogel, which formed a rigid gel structure and exhibited excellent stability at pH 2, maintained small gel particles even during the stomach phase. This suggests that the Saemimyeon-based filled hydrogel can withstand the sequential deformation caused by continuous agitation, effectively inhibiting the release of VD within lipid droplets that occurs during in vitro digestion. In the Saemimyeon-based filled hydrogel, the strong gel matrix formed through rapid retrogradation may act as a protective barrier, effectively hindering VD in lipid droplets from being exposed to digestive enzymes in digestive fluid (Guo et al., 2014). In contrast, the Dodamssal-based filled hydrogel, which formed a relatively weak gel due to incomplete gelatinization with unswollen starch granules, allowed VD in the emulsion to be exposed to digestive fluid due to the release of the emulsion and water. As a result, the VD retention rate of the Dodamssal-based filled hydrogel was significantly lower than that of the Saemimyeon-based filled hydrogel. The Mirchal-based filled hydrogel did not undergo retrogradation, even after overnight storage, and formed a gel with low viscosity and elasticity (Figs. 2B and C). Consequently, it was easily dissolved and destroyed in the digestive fluid, which may have increased VD oxidation due to the increased exposure of lipid droplets during the digestion process. In summary, the use of filled hydrogels with high elasticity prevented the destruction of the gel matrix during digestion and controlled the release rate of oil. Therefore, filled hydrogels may play a role in increasing the accessibility of VD by minimizing lipid digestion through interference with enzyme diffusion.

In conclusion, the encapsulation performance of VD by rice flour-based filled hydrogel was closely related to the properties of the gel network, including the amylose content and the gelatinization properties of the rice flour used. The filled hydrogel produced with Saeilmi, which has an amylose content of 19.1%, displayed less retrogradation and higher freeze–thaw stability, as a gel was formed with lower elasticity compared to the Saemimyeon-based filled hydrogel. Mirchal (< 5% amylose content) has a significantly higher water solubility index and did not form a rigid hydrogel. Dodamssal, with a high amylose content of > 40% and a high resistant starch content, had a high gelatinization temperature and exhibited weak gel network formation. Due to these properties, the filled hydrogel prepared with Dodamssal resulted in gel shrinkage, but the VD retention rate was significantly higher than that of the emulsion system. The filled hydrogel prepared from Saemimyeon rice flour containing 25.5% amylose had a high G' value and strong elasticity. As a result, not only the stability of VD but also the retention rate of VD during in vitro digestion was shown to be the highest. Consequently, the formation of a solid-like gel structure by starch gelatinization of rice flour and retrogradation via hydrogen bonding proved to be effective compared to the emulsion alone, as a delivery system that can improve the stability of encapsulated VD. These results imply that the amylose content of the rice flour used in filled hydrogel production can regulate the physicochemical properties of the gel matrix and enhance the stability of functional materials. The results of this study suggest the potential utility of rice flour as a promising material for the development of hydrogels tailored for functional food delivery.

## Supplementary Information

Below is the link to the electronic supplementary material.

Supplementary file1 (DOCX 24 KB)

## References

[CR1] AACC. Approved methods of the American Association of Cereal Chemists, 10th ed. methods 61–02.01. American Association of Cereal Chemists. St. Paul, MN, USA (2000)

[CR2] Biduski B, de Silva WMF, Colussi R, El Halal SLM, Lim LT, Dias ÁRG, da Rosa Zavareze E. Starch hydrogels: The influence of the amylose content and gelatinization method. International Journal of Biological Macromolecules. 113: 443-449 (2018) 29486261 10.1016/j.ijbiomac.2018.02.144

[CR3] Calero N, Muñoz J, Cox PW, Heuer A, Guerrero A. Influence of chitosan concentration on the stability, microstructure and rheological properties of O/W emulsions formulated with high-oleic sunflower oil and potato protein. Food Hydrocolloids. 30: 152-162 (2013).

[CR4] Chang Y, McClements DJ. Characterization of mucin–lipid droplet interactions: Influence on potential fate of fish oil-in-water emulsions under simulated gastrointestinal conditions. Food Hydrocolloids. 56: 425-433 (2016).

[CR5] Chumsri P, Panpipat W, Cheong LZ, Chaijan M. Formation of intermediate amylose rice starch–lipid complex assisted by ultrasonication. Foods. 11: 2430 (2022). 36010430 10.3390/foods11162430 PMC9407459

[CR7] Coviello T, Matricardi P, Marianecci C, Alhaique F. Polysaccharide hydrogels for modified release formulations. Journal of Controlled Release. 119: 5-24 (2007). 17382422 10.1016/j.jconrel.2007.01.004

[CR8] da Rosa Zavareze E, Mello El Halal SL, de los Santos DG, Helbig E, Pereira JM, Guerra Dias AR. Resistant starch and thermal, morphological and textural properties of heat‐moisture treated rice starches with high‐, medium‐, and low‐amylose content. Starch‐Stärke. 64: 45–54 (2012)

[CR9] Ezhilarasi P, Karthik P, Chhanwal N, Anandharamakrishnan C. Nanoencapsulation techniques for food bioactive components: a review. Food Bioprocess Technology. 6: 628-647 (2013).

[CR10] Farooq MA, Murtaza MA, Aadil RM, Arshad R, Rahaman A, Siddique R, Hassan S, Akhtar HMS, Manzoor MF, Karrar E. Investigating the structural properties and in vitro digestion of rice flours. Food Science & Nutrition. 9: 2668-2675 (2021). 34026080 10.1002/fsn3.2225 PMC8116841

[CR11] Fu ZQ, Wang LJ, Li D, Zhou YG, Adhikari B. The effect of partial gelatinization of corn starch on its retrogradation. Carbohydrate Polymers. 97: 512-517 (2013). 23911478 10.1016/j.carbpol.2013.04.089

[CR12] Guo Q, Ye A, Lad M, Dalgleish D, Singh H. Effect of gel structure on the gastric digestion of whey protein emulsion gels. Soft Matter. 10: 1214-1223 (2014). 24652237 10.1039/c3sm52758a

[CR13] Guo Q, Ye A, Lad M, Ferrua M, Dalgleish D, Singh H. Disintegration kinetics of food gels during gastric digestion and its role on gastric emptying: an in vitro analysis. Food Function*.* 6: 756-764 (2015). 25562505 10.1039/c4fo00700j

[CR14] Hemery YM, Fontan L, Moench-Pfanner R, Laillou A, Berger J, Renaud C, Avallone S. Influence of light exposure and oxidative status on the stability of vitamins A and D3 during the storage of fortified soybean oil. Food Chemistry. 184: 90-98 (2015). 25872430 10.1016/j.foodchem.2015.03.096

[CR15] Hong EM, Rho SJ, Kim U, Kim YR. Physicochemical properties and freeze–thaw stability of rice flour blends among rice cultivars with different amylose contents. Food Science and Biotechnology. 30: 1347-1356 (2021). 34721930 10.1007/s10068-021-00989-7 PMC8519984

[CR16] Houjyo K, Sugiyama J, Kokawa M, Fujita K, Yuge W, Nozaki R, Itoh T. Effect of emulsification on the physical properties of high-amylose rice gel. Food Science and Technology Research. 23: 221-228 (2017).

[CR17] Kang J, Kim YH, Choi SJ, Rho SJ, Kim YR. Improving the stability and curcumin retention rate of curcumin-loaded filled hydrogel prepared using 4αgtase-treated rice starch. Foods. 10: 150 (2021). 33450818 10.3390/foods10010150 PMC7828239

[CR18] Kim D, Jung Y, Rho SJ, Kim YR. Improved stability and in vitro bioavailability of β-carotene in filled hydrogel prepared from starch blends with different granule sizes. Food Hydrocolloids. 139: 108546 (2023).

[CR19] Kong Y, Hay JN. The measurement of the crystallinity of polymers by DSC. Polymer. 43: 3873-3878 (2002).

[CR20] Li E, Yang X, Li C. Combined effects of starch fine molecular structures and storage temperatures on long-term rice amylopectin retrogradation property. International Journal of Biological Macromolecules. 201: 458-467 (2022). 35063484 10.1016/j.ijbiomac.2022.01.092

[CR21] Light K, Karboune S. Emulsion, hydrogel and emulgel systems and novel applications in cannabinoid delivery: A review. Critical Reviews in Food Science and Nutrition. 62: 8199-8229 (2022). 34024201 10.1080/10408398.2021.1926903

[CR22] Liu F, Ma C, Zhang R, Gao Y, McClements DJ. Controlling the potential gastrointestinal fate of β-carotene emulsions using interfacial engineering: Impact of coating lipid droplets with polyphenol-protein-carbohydrate conjugate. Food Chemistry. 221: 395-403 (2017). 27979220 10.1016/j.foodchem.2016.10.057

[CR23] Liu K, Kong XL, Li QM, Zhang HL, Zha XQ, Luo JP. Stability and bioavailability of vitamin D_3_ encapsulated in composite gels of whey protein isolate and lotus root amylopectin. Carbohydrate Polymers. 227: 115337 (2020). 31590880 10.1016/j.carbpol.2019.115337

[CR24] Ma Z, Ma M, Zhou D, Li X, Hu X. The retrogradation characteristics of pullulanase debranched field pea starch: Effects of storage time and temperature. International Journal of Biological Macromolecules. 134: 984-992 (2019). 31082424 10.1016/j.ijbiomac.2019.05.064

[CR25] Mahmoodani F, Perera CO, Fedrizzi B, Abernethy G, Chen H. Degradation studies of cholecalciferol (vitamin D_3_) using HPLC-DAD, UHPLC-MS/MS and chemical derivatization. Food Chemistry. 219: 373-381 (2017). 27765240 10.1016/j.foodchem.2016.09.146

[CR26] Majeed H, Antoniou J, Hategekimana J, Sharif HR, Haider J, Liu F, Ali B, Rong L, Ma J, Zhong F. Influence of carrier oil type, particle size on in vitro lipid digestion and eugenol release in emulsion and nanoemulsions. Food Hydrocolloids. 52: 415-422 (2016).

[CR27] Mekala S, Silva EK, Saldaña MD. Ultrasound-assisted production of emulsion-filled pectin hydrogels to encapsulate vitamin complex: Impact of the addition of xylooligosaccharides, ascorbic acid and supercritical CO_2_ drying. Innovative Food Science & Emerging Technologies. 76: 102907 (2022).

[CR28] Mun S, Kim YR, McClements DJ. Control of β-carotene bioaccessibility using starch-based filled hydrogels. Food Chemistry. 173: 454-461 (2015). 25466045 10.1016/j.foodchem.2014.10.053

[CR29] Puncha-arnon S, Pathipanawat W, Puttanlek C, Rungsardthong V, Uttapap D. Effects of relative granule size and gelatinization temperature on paste and gel properties of starch blends. Food Research International. 41: 552-561 (2008).

[CR30] Reis AV, Guilherme MR, Moia TA, Mattoso LH, Muniz EC, Tambourgi EB. Synthesis and characterization of a starch‐modified hydrogel as potential carrier for drug delivery system. Journal of Polymer Science Part A: Polymer Chemistry. 46: 2567-2574 (2008).

[CR31] Salvia-Trujillo L, Qian C, Martín-Belloso O, McClements DJ. Influence of particle size on lipid digestion and β-carotene bioaccessibility in emulsions and nanoemulsions. Food Chemistry. 141: 1472-1480 (2013). 23790941 10.1016/j.foodchem.2013.03.050

[CR32] Sasaki T, Yasui T, Matsuki J. Effect of amylose content on gelatinization, retrogradation, and pasting properties of starches from waxy and nonwaxy wheat and their F1 seeds. Cereal Chemistry. 77: 58-63 (2000).

[CR33] Sharma M, Yadav DN, Singh AK, Tomar SK. Effect of heat-moisture treatment on resistant starch content as well as heat and shear stability of pearl millet starch. Agricultural Research. 4: 411-419 (2015).

[CR34] Temova Rakuša Ž, Pišlar M, Kristl A, Roškar R. Comprehensive stability study of vitamin D3 in aqueous solutions and liquid commercial products. Pharmaceuticals. 13: 617 (2021). 10.3390/pharmaceutics13050617 PMC8147103 33922975

[CR35] Tian Y, Yang N, Li Y, Xu X, Zhan J, Jin Z. Potential interaction between β-cyclodextrin and amylose–lipid complex in retrograded rice starch. Carbohydrate Polymers. 80: 581-584 (2010).

[CR36] Yuan R, Thompson D. Freeze‐thaw stability of three waxy maize starch pastes measured by centrifugation and calorimetry. Cereal Chemistry. 75: 571-573 (1998).

[CR37] Zhang X, Guo D, Xue J, Yanniotis S, Mandala I. The effect of salt concentration on swelling power, rheological properties and saltiness perception of waxy, normal and high amylose maize starch. Food Function. 8: 3792-3802 (2017). 28960010 10.1039/c7fo01041a

[CR38] Zhou X, Baik BK, Wang R, Lim ST. Retrogradation of waxy and normal corn starch gels by temperature cycling. Journal of Cereal Science. 51: 57-65 (2010).

[CR39] Zhu LJ, Liu QQ, Wilson JD, Gu MH, Shi YC. Digestibility and physicochemical properties of rice (Oryza sativa L.) flours and starches differing in amylose content. Carbohydrate Polymers. 86: 1751-1759 (2011).

