# Supplementary material for: Rice flour-based filled hydrogel: an effective vitamin D encapsulation system as influenced by rice flour variety
Source: Food Sci Biotechnol. 2025 Jan 7;34(7):1617–29. doi: 10.1007/s10068-024-01806-7 (PMC11929660; doi:10.1007/s10068-024-01806-7)
Supplement: Supplementary file 1 — Supplementary file1 (DOCX 24 KB) [file 10068_2024_1806_MOESM1_ESM.docx]

Supplementary data

Table S1. Rheological behavior parameters of rice flour-based filled hydrogels

| Filled hydrogels | Power-law parameters^1^ | | |  | Dynamic rheological parameters^2^ | | |
| --- | --- | --- | --- | --- | --- | --- | --- |
|  | *K* (Pa) | *n* | R^2^ |  | G' (Pa) | G'' (Pa) | tan δ |
| Dodamssal | 8.57±4.41^b^ | 1.60±0.26^a^ | 0.94±0.03^a^ |  | 11.08±4.45^c^ | 19.91±8.13^b^ | 1.80±0.24^a^ |
| Saemimyeon | 349.16±51.38^a^ | 0.07±0.01^c^ | 0.85±0.03^b^ |  | 356.67±125.17^a^ | 30.57±7.99^a^ | 0.09±0.02^e^ |
| Saeilmi | 44.83±6.42^b^ | 0.30±0.05^c^ | 0.92±0.07^bc^ |  | 47.69±10.10^bc^ | 17.38±1.61^bc^ | 0.37±0.04^d^ |
| Mirchal | 8.30±1.30^b^ | 0.87±0.04^b^ | 0.99±0.01^a^ |  | 9.28±1.28^c^ | 8.98±1.11^c^ | 0.97±0.05^b^ |

^1^ Rheological model is presented in Eq. (1). *K* is the consistency index, *n* is the flow behavior index, and R^2^ is the determination coefficient.

^2^ Rheological properties of rice flour-based filled hydrogels at 1 Hz. G’ and G” are the storage (G′) and loss moduli (G″), respectively, and tan δ is calculated as G″/G′.

The results are expressed as mean ± standard deviation. Different letters in the same columns are significantly different (*p* < 0.05).
